# How to guide PCI? A network meta-analysis

**DOI:** 10.1097/MD.0000000000020168

**Published:** 2020-05-15

**Authors:** Jun Pang, Liwen Ye, Qingwei Chen

**Affiliations:** Department of Geriatric Cardiology, the Second Affiliated Hospital of Chongqing Medical University, Chongqing, China.

**Keywords:** coronary angiography, fractional flow reserve, instantaneous wave-free ratio, intravascular, optical coherence tomography, ultrasound

## Abstract

**Background:**

Traditional coronary angiography (CA) as a main technique has been used to determine the coronary artery anatomy and guide percutaneous coronary intervention (PCI). We mainly focused on whether the new techniques could improve the patients’ mortality, major adverse cardiovascular events (MACEs), and myocardial infarction.

**Methods:**

For the network meta-analysis, we searched the trials of different PCI guidances from MEDLINE, Current Contents Connect, Google Scholar, EMBASE, Cochrane Library, PubMed, Science Direct, and Web of Science. The last search date was December 10, 2018.

**Results:**

The analyses of all results found that there was no significant difference in mortality among the groups. Randomized clinical trials (RCT) analysis showed that intravascular ultrasound (IVUS)-guided PCI was significantly superior to CA, fractional flow reserve, instantaneous wave-free ratio, optical coherence tomography. However, CA, fractional flow reserve, instantaneous wave-free ratio, and optical coherence tomography showed no difference in reducing mortality. The analyses of all results found that there was no significant difference in the incidence of MACEs among the groups. RCTs analysis showed that IVUS-guided PCI was significantly superior to CA, but there was no significant difference among the other groups. The analyses of all results or RCTs showed that there was no significant difference in myocardial infarction incidence among the groups.

**Conclusion:**

IVUS-guided PCI is an effective method to decrease all-cause death MACEs.

## Abstract 2

Supplemental Digital Content is available in the text

## 1 Introduction

Coronary angiography (CA) is a common diagnostic tool for ischemic heart disease. However, the visual assessment of lesion severity does not always reflect the hemodynamics of coronary artery stenosis.^[1,2,3,4]^ With the research constantly deepens, people gradually realize that coronary artery lesions should not be limited to anatomical stenosis, the functional changes induced by stenosis should be paid more attention.

Fractional flow reserve (FFR) is a ratio of distal and proximal pressures at time of maximal coronary artery congestion induced by vasodilators (usually adenosine). FFR has been used to guide coronary artery reconstruction for more than a decade.^[5]^ Some randomized clinical trials (RCTs) have validated that FFR is effective in guiding deferring invasive treatment for non significant lesions compared with angiography.^[6,7,8,9,10]^ The necessity of vasodilators assessing the severity of stenosis has been questioned, especially in patients with microcirculation disturbance, such as acute coronary syndrome or kidney disease.^[11,12,13]^ Secondly, these vasodilators have side effects.^[13]^ Instantaneous wave-free ratio (iFR) is a physiological index to evaluate the degree of stenosis in recent years. Unlike FFR, vasodilators are not necessary for iFR to assess stenosis. It represents the pressure gradient of stable coronary artery stenosis with low microvascular resistance at diastole (wave-free period).^[14]^ Two large RCTs proved that iFR was not inferior to FFR in guiding revascularization.

Due to the limitations of CA in percutaneous coronary intervention (PCI), intravascular imaging techniques such as intravascular ultrasound (IVUS), optical coherence tomography (OCT) are more and more widely used in PCI.^[15]^

IVUS and OCT can accurately assess vascular stenosis, plaque morphology and has high spatial resolution, which is of great value to optimize the results of PCI.^[16]^ Compared with the simple CA-guided PCI, IVUS-guided PCI can better expand the stents and improve the survival rate.^[17,18]^ The resolution of OCT is superior to that of IVUS.^[19]^ However, the penetration depth of OCT is limited in many cases. Compared with angiography or IVUS, the risk-benefit effect of OCT is uncertain in routine clinical practice.^[20,21]^

Traditional CA as a main technique, has been used to determine the coronary artery anatomy and guide PCI. But this method has several disadvantages. We mainly focused whether the new techniques could improve the patients’ mortality, major adverse cardiovascular events (MACEs) and myocaridial infarction (MI).

## 2 Methods

### 2.1 Search strategy and selection criteria

For the network meta-analysis (NMA), we searched the trials of different PCI guidances from MEDLINE, Current Contents Connect, Google Scholar, EMBASE, Cochrane Library, PubMed, Science Direct, and Web of Science. Search MeSH terms included “percutaneous coronary intervention”, “PCI”, “coronary angiography”, “fractional flow reserve”, “FFR”, “instantaneous free-wave ratio”, “iFR”, “interventional ultrasound”, “intravascular ultrasound”, “IVUS”, “intracoronary ultrasound”, “ICUS”, “optical coherence tomography”, “OCT”, “optical frequency domain”, and “OFDI”. The last search date was December 10, 2018.

### 2.2 Study selection

#### 2.2.1 Inclusion criteria

a. The subjects were patients undergoing PCI.
b. The randomized controlled trails and non-randomized prospective trails as well as non-randomized retrospective trails were conducted for different PCI methods.
c. The intervening measures included CA, FFR, iFR, IVUS, and OCT.
d. At least 1 of the data was reported in this paper (namely major adverse cardiac events, or MACE, all-cause mortality, myocardial infarction, or MI).

#### 2.2.2 Exclusion criteria

The papers only with abstracts, meetings, and protocols were excluded from this paper.

### 2.3 Data extraction

Two reviewers (LY, JP) screened the title and abstract of the paper as well as the potential related full-text articles. Then the data were extracted from the selected studies, including study characteristics, patient characteristics, and outcomes. End-point definition included all-cause mortality, MI, repeat revascularization, stent thrombosis, and MACEs. MACE included all-cause mortality, MI, repeat target vessel revascularization, and stent thrombosis.

In view of the impacts of RCTs, prospective and retrospective studies on the outcome, all studies were analyzed first and RCTs data were analyzed separately.

### 2.4 Quality assessment

**Figure 1:**
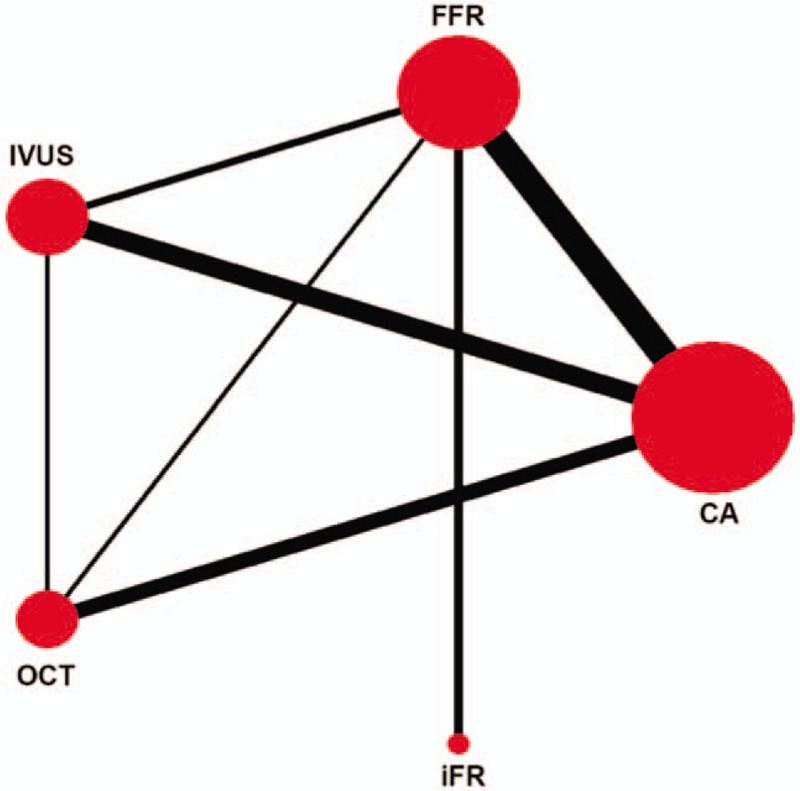
Network showing the direct comparisons of the effect on mortality. The numbers of patients and directly compared trials are shown. (CA = coronary angiography, FFR = fractional flow reserve, iFR = instantaneous wave-free ratio, IVUS = intravascular ultrasound, OCT = optical coherence tomography).

Two authors (LY and XY) reviewed the main reports and supplementary materials independently. Then the authors extracted the relevant data from the articles using the Cochrane risk of bias tool (http://handbook.cochrane.org). If there were difference in the process of evaluating the methodological quality, the team members (QC, LY, JP, and XY) would negotiate to resolve them (see Fig. 1, Supplemental Content, which showed the risk of bias of the included trials).

### 2.5 Data synthesis and analysis

First, the pairwise meta-analysis was performed by using R version 3.4.1 software (http://www.r.project.org). The continuous variables were expressed using mean difference and 95% confidence interval. Heterogeneity test using I^2^-statistics and *P*-value showed that I^2^ < 25% was mildly heterogeneous, I^2^ 25% to 50% was moderately heterogeneous, and I^2^ > 50% was highly heterogeneous.^[22]^ If there was enough homogeneity between the results of the study, Mantel–Haenzel fixed effects model should be used for combined analysis; If there was heterogeneity between the results of the study, DerSimonian–Laird random effects model should be used.

NMA combined direct and indirect comparative evidence and provided the estimated maximum statistical power for all related therapies.^[23]^ Based on Bayesian framework, the meta-analysis was performed using GeMTC version 0.14.3 (http://drugis.org/sofware/addis1/), JAGS-4.2.0 (http://mcmc-jags.sourceforge.net/), and R version 3.4.1 (http://www.r.project.org). The GeMTC, rjags software packages in this study were searched from R software. The direct comparison between different interventions was presented by drawing a network diagram. The continuous variables were expressed as efficacy statistics using mean difference and 95% confidence interval. All results pertained to 10,000 tuning iterations and 200,000 Markov Chain Monte Carlo cycles. Three Monte Carlo Markov chains were obtained, so as to evaluate the convergence of the results. The consistency of network results was evaluated by comparing pairwise meta-analysis. The inconsistency between direct comparison and indirect comparison results was measured by node splitting value. *P* < .05 was considered that the inconsistency was significant and the direct comparison results was preferred. The ranking probabilities were used to assess the impact of different PCI guidance measures on end-point events.^[23]^ Also the rank probability plot was drawn.

### 2.6 Ethical Statement

As this meta-analysis was based on previously published studies, ethical approval was not necessary.

## 3 Results

A total of 3021 relevant articles were searched. Finally, a total of 67 potentially eligible articles were selected. Of these, 49 were excluded because the trials were on stent implantation (n = 29), duplicate trials (n = 9), non-controlled trials (n = 6), or patients with MI (n = 5). As a result, 49 eligible articles were excluded. Finally, 18 studies were enrolled in our meta-analysis (see Fig. 2, Supplemental Content, which showed the flow diagram of this article). A flow diagram depicting this disposition of the studies was constructed according to the Preferred Reporting Items for Systematic Reviews and Meta-Analyses Statement.^[24]^

Eighteen studies were included in this NMA that involved 62,197 patients, so as to assess the impacts of various methods guiding PCI on mortality. These studies included 10 RCTs (7822 patients), 4 prospective studies (1759 patients) and 4 retrospective studies (52,616 patients).

### 3.1 Study characteristics

The study characteristics are listed in Supplemental Content (see Table 1, Supplemental Content, which show characteristics of studies). The number of trials that directly compared the treatments and the number of patients involved in each direct comparison are depicted schematically in Figure 1.

### 3.2 All-cause death

Eighteen studies were included in this NMA, so as to assess the impacts of various methods guiding PCI on mortality. These studies included 10 RCTs, 4 prospective studies, and 4 retrospective studies.

Most pairwise meta-analysis heterogeneity results showed I^2^ > 50%. DerSimonian–Laird random effects model was selected. Pairwise meta-analysis results were closely matched with those of NMA results (see Fig. 3, and Fig. 4, Supplemental Content, which showed the pairwise meta-analysis and heterogeneity comparing different kinds of guidance for PCI on mortality). After multiple iterations, the convergence graph results showed that the convergence of each group was good (see Fig. 5, and Fig. 6, Supplemental Content, which showed the convergence graph of guidance for PCI on mortality). The analyses of all results found that there was no significant difference in mortality among the groups (Table 1). RCT analysis showed that IVUS-guided PCI was significantly superior to CA, FFR, iFR, OCT. However, CA, FFR, iFR, and OCT showed no difference in reducing mortality (Table 1). The ranking result was shown in Supplemental Content (see Fig. 7, Supplemental Content, which showed the rank probability on mortality).

**Table 1:**
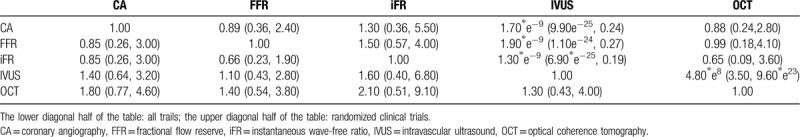
Relative efficacy of guidance for percutaneous coronary intervention on mortality, as determined by the network meta-analysis.

### 3.3 MACEs

Eighteen studies were included in this NMA, so as to assess the impacts of various methods guiding PCI on MACEs. These studies included 10 RCTs, 4 prospective studies, and 4 retrospective studies.

The data were analyzed using the DerSimonian–Laird random effects model. Pairwise meta-analysis results were closely matched with the NMA results (see Fig. 8, and Fig. 9, Supplemental Content, which showed the pairwise meta-analysis and heterogeneity comparing different kinds of guidance for PCI on MACEs). After multiple iterations, the results of convergence graph suggested that the convergence of each group was good (see Fig. 10, and Fig. 11, Supplemental Content, which showed the convergence graph of guidance for PCI on MACEs). The analyses of all results found that there was no significant difference in the incidence of MACEs among the groups (Table 2). RCT analysis showed that IVUS IVUS-guided PCI was significantly superior to CA, but there was no significant difference among the other groups (Table 2). The ranking results was shown in Supplemental Content (see Fig. 12, Supplemental Content, which showed the rank probability on MACEs). The probability of top IVUS ranking was superior to that of other groups, while the probability of low-ranking CA was maximum.

**Table 2:**
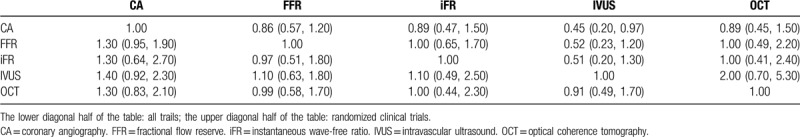
Relative efficacy of guidance for percutaneous coronary intervention on major adverse cardiovascular events, as determined by the network meta-analysis.

### 3.4 MI

Twelve studies were included in this NMA, so as to assess the impacts of various methods guiding PCI on MACEs. These studies included 8 RCTs, 1 prospective study, and 3 retrospective studies.

The data were analyzed using the DerSimonian–Laird random effects model. Pairwise meta-analysis results were closely matched with the NMA results (see Fig. 13, and Fig. 14, Supplemental Content, which showed the pairwise meta-analysis and heterogeneity comparing different kinds of guidance for PCI on MI). After multiple iterations, the results of convergence graph suggested that the convergence of each group was good (see Fig. 15, and Fig. 16, Supplemental Content, which showed the convergence graph of guidance for PCI on MI). The analyses of all results or RCTs showed that there was no significant difference in MI incidence among the groups (Table 3). The probability of top OCT ranking was superior to that of other groups, while the probability of low-ranking iFR was maximum (see Fig. 17, Supplemental Content, which showed the rank probability on MI). RCT analysis showed that there was no significant difference among the groups (Table 3).

**Table 3:**
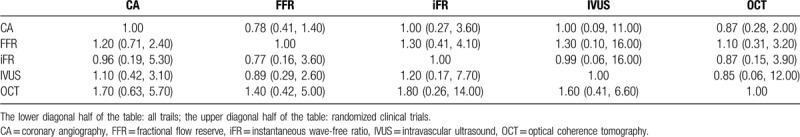
Relative efficacy of guidance for percutaneous coronary intervention on myocardial infarction, as determined by the network meta-analysis.

## 4 Discussion

Although CA has been widely used to evaluate coronary artery disease, it cannot always accurately predict the functional significance of coronary artery stenosis.^[25]^ Therefore, the other diagnostic methods are recommended to comprehensively evaluate the clinical effects of coronary artery disease.

FFR is an invasive index for evaluating the functional significance of coronary artery stenosis.^[26]^ In addition, FFR-guided PCI not only can improve the clinical efficacy,^[27]^ but also can reduce the unnecessary PCI compared with angiography-guided PCI, thus saving the cost. FFR can accurately determine whether the coronary artery lesion or segment can cause myocardial ischemia and is not affected by blood pressure, heart rate and systemic hemodynamics.^[5,28,29]^ Previous meta-analysis has found that FFR-guided PCI can reduce the incidence of MACEs in patients compared with CA-guided PCI. FFR-guided PCI can significantly reduce the incidence of MACEs of patients. The 5-year follow-up results published in FAME study also confirms the safety of FFR-guided PCI.^[30]^

IFR is a new non-vasodilator index to measure the severity of coronary artery stenosis. It represents and the trans-lesion pressure ratio on both sides of the lesion at a specific period of baseline diastole when distal resistance is lowest and stable.^[31]^ IFR does not depend on vasodilators.^[14,32]^ It can simplify the assessment of intracoronary function with lower cost, fewer patient discomfort, and shorter operation time than FFR.

Both FFR and iFR are the detection of lesion vascular pressure, which belongs to functional detection. Whereas, IVUS and OCT are the intracoronary imaging techniques for the anatomical/morphological evaluation of coronary artery lesions, which belongs to the detection of coronary artery structure.

IVUS transmits miniaturized ultrasound probe into coronary artery by catheterization technology and emits ultrasonic pulse at 360° to the vascular wall, which converts the reflected acoustic wave from vascular wall tissue into an image of a cross-section of the coronary artery through electrical signals. Different types of tissue characteristics can be distinguished from IVUS images according to the supersonic absorption and reflected echo signal induced by density variation of vascular wall tissues. Some studies reported a significant correlation between the minimum lumen area measured by IVUS and FFR.^[33,34]^ The venule-derived MLA is mainly associated with the incidence of MACEs caused by vascular restenosis.^[35]^

The operating principle of OCT is similar to that of IVUS. It adopts near-infrared ray instead of acoustic wave to detect the tissue structure. The same light source is divided into 2 parts. Then optical interference is realized from the return stroke reflected from vascular tissue and the mobile interface, so as to form real-time coronary artery tomography. The time difference of reflective optics interference wave reflects the distance of the moving interface, which is used as a standard for measuring tissue spacing. The wave length of optical wave is very short, so the resolution of OCT is very high. However, the penetrating power of the corresponding OCT to the vessel wall is limited. The radius of visual field is only 3 to 4 mm and the depth of fluoroscopy tissue is only 2 to 2.5 mm. It cannot be effectively applied to the imaging of large coronary artery (diameter > 4 mm). In addition, a large number of red blood cells in blood can induce the infrared light scattering, the balloon catheter is required to block the proximal coronary artery and the target vessel is washed with normal saline in order to obtain the clear and stable images, which obviously limits the application of OCT in opening lesion and left main coronary artery lesion. The new generation of OCT imaging system adopts scanning laser as light source, which has faster imaging speed, stronger penetration, higher resolution, and no blood flow blocking.

The previous report showed that OCT was more effective to diagnose the coronary artery stenosis than IVUS hemodynamically.^[33]^ Although IVUS is superior to OCT in guiding PCI, which may be related to the disadvantage of OCT, OCT can better display the true lumen interface than IVUS, indicating that OCT-derived MLA may also have the potential to predict the risk of MACE.^[36]^ With the improvement of OCT imaging technology, it is possible to surpass IVUS in guiding PCI.

## 5 Conclusion

IVUS-guided PCI is an effective method to decrease all-cause death MACEs.

## Acknowledgments

We gratefully acknowledge the assistance of our colleagues during the writing of this paper and the helpful advice of Li Wang and Wei Deng.

## Author contributions

**Conceptualization:** Qingwei Chen.

**Data curation:** Jun Pang, Liwen Ye.

**Formal analysis:** Jun Pang, Liwen Ye.

**Methodology:** Jun Pang, Liwen Ye.

**Project administration:** Qingwei Chen.

**Software:** Jun Pang, Liwen Ye.

**Supervision:** Qingwei Chen.

**Writing – original draft:** Jun Pang.

**Writing – review & editing:** Liwen Ye.

## Supplementary Material

Supplemental Digital Content

## Supplementary Material

Supplemental Digital Content

## Supplementary Material

Supplemental Digital Content

## Supplementary Material

Supplemental Digital Content

## Supplementary Material

Supplemental Digital Content

## Supplementary Material

Supplemental Digital Content

## Supplementary Material

Supplemental Digital Content

## Supplementary Material

Supplemental Digital Content

## Supplementary Material

Supplemental Digital Content

## Supplementary Material

Supplemental Digital Content

## Supplementary Material

Supplemental Digital Content

## Supplementary Material

Supplemental Digital Content

## Supplementary Material

Supplemental Digital Content

## Supplementary Material

Supplemental Digital Content

## Supplementary Material

Supplemental Digital Content

## Supplementary Material

Supplemental Digital Content

## Supplementary Material

Supplemental Digital Content

## Supplementary Material

Supplemental Digital Content

## Supplementary Material

Supplemental Digital Content
