# Supplementary material for: How to guide PCI? A network meta-analysis
Source: Medicine (Baltimore). 2020 May 15;99(20):e20168. doi: 10.1097/MD.0000000000020168 (PMC7253719; doi:10.1097/MD.0000000000020168)

**Figures**

**Figure 1 A.** Risk of bias summary. Judgements about each bias item for each study. **B.** Risk of bias graph: review authors' judgements (Low, Unclear and High) about each risk of bias item presented as percentages across all included studies.


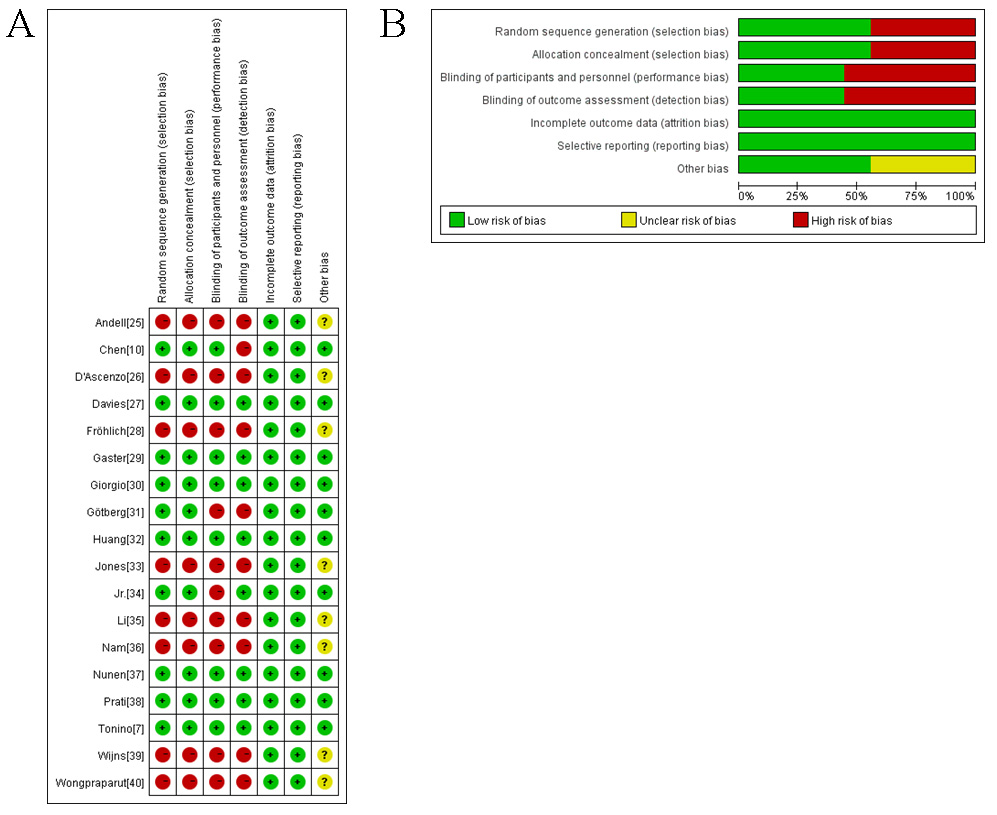

Supplement: Supplemental Digital Content [file medi-99-e20168-s001.docx]
