# Supplementary material for: How to guide PCI? A network meta-analysis
Source: Medicine (Baltimore). 2020 May 15;99(20):e20168. doi: 10.1097/MD.0000000000020168 (PMC7253719; doi:10.1097/MD.0000000000020168)

Figure 12. Rank probability plots of the major adverse cardiovascular events results of the network meta-analysis. As the rank decreases, the bar becomes lighter. (A: all trials; B: randomized clinical trials)


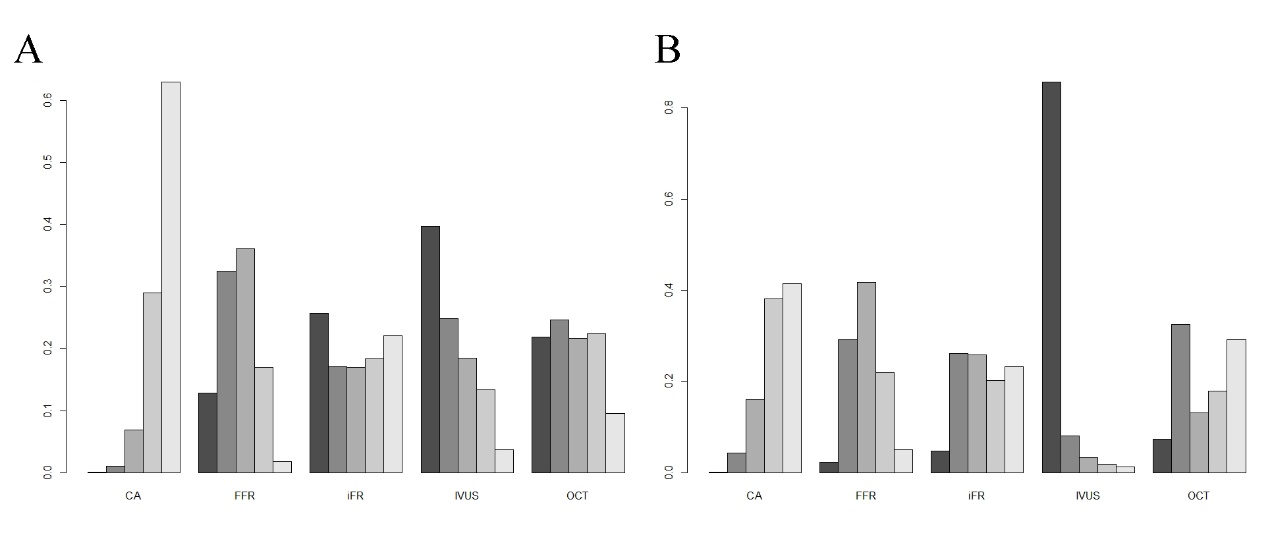

Supplement: Supplemental Digital Content [file medi-99-e20168-s012.docx]
