# Supplementary material for: How to guide PCI? A network meta-analysis
Source: Medicine (Baltimore). 2020 May 15;99(20):e20168. doi: 10.1097/MD.0000000000020168 (PMC7253719; doi:10.1097/MD.0000000000020168)

Figure 16. Convergence graph of guidance for percutaneous coronary intervention on myocardial infarction (randomized clinical trials).


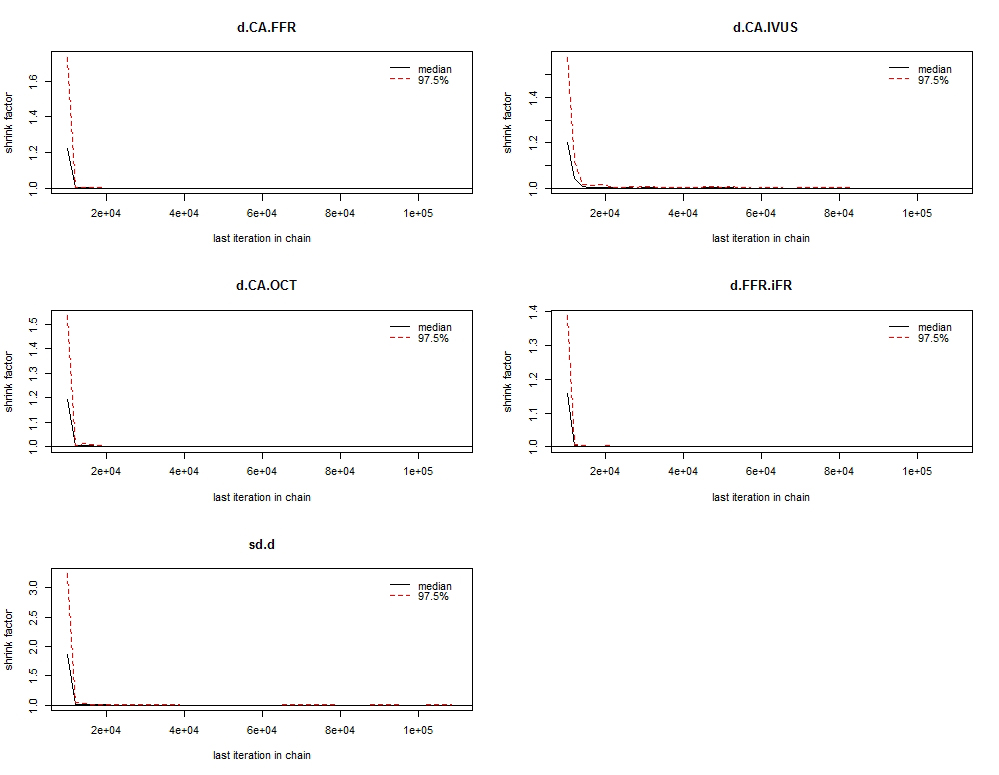

Supplement: Supplemental Digital Content [file medi-99-e20168-s016.docx]
