# Supplementary material for: How to guide PCI? A network meta-analysis
Source: Medicine (Baltimore). 2020 May 15;99(20):e20168. doi: 10.1097/MD.0000000000020168 (PMC7253719; doi:10.1097/MD.0000000000020168)
Supplement: Supplemental Digital Content [file medi-99-e20168-s018.docx]

Table 1. Baseline characteristics of the trials that were included in the network meta-analysis. (C, coronary angiography; FFR, fractional flow reserve; iFR, instantaneous wave-free ratio; IVUS, intravascular ultrasound; M, method; RCT, randomized clinical trials; OCT, optical coherence tomography; S, sample size.)

| Author | Year | Study | RCT | M1 | S1 | M2 | S2 | M3 | S3 |
| --- | --- | --- | --- | --- | --- | --- | --- | --- | --- |
| Andell^1^ | 2017 | - | prospective | CA | 340 | IVUS | 340 |  |  |
| Chen^2^ | 2015 | ILUMIEN I | prospective | CA | 137 | OCT | 165 |  |  |
| D'Ascenzo^3^ | 2017 | - | prospective | OCT | 285 | FFR | 355 |  |  |
| Davies^4^ | 2017 | DEFINE-FLAIR | RCT | iFR | 1147 | FFR | 1179 |  |  |
| Fröhlich^5^ | 2014 | - | retrospective | CA | 37090 | FFR | 2767 | IVUS | 1831 |
| Gaster^6^ | 2003 | MUSIC | RCT | IVUS | 54 | CA | 54 |  |  |
| Giorgio^7^ | 2013 | - | RCT | OCT | 40 | CA | 40 |  |  |
| Götberg^8^ | 2017 | iFR-SWEDEHEART | RCT | iFR | 1012 | FFR | 1007 |  |  |
| Huang^9^ | 2017 | - | RCT | FFR | 101 | CA | 105 |  |  |
| Jones^10^ | 2018 | Pan-London | retrospective | CA | 1134 | OCT | 1134 | IVUS | 1125 |
| Jr.^11^ | 2015 | MOZART | RCT | IVUS | 41 | CA | 42 |  |  |
| Li^12^ | 2012 | - | retrospective | CA | 6268 | FFR | 1090 |  |  |
| Nam^13^ | 2010 | - | retrospective | FFR | 83 | IVUS | 94 |  |  |
| Nunen^14^ | 2015 | FAME | RCT | CA | 496 | FFR | 509 |  |  |
| Prati^15^ | 2012 | CLI-OPCI | RCT | OCT | 335 | CA | 335 |  |  |
| Tonino^16^ | 2009 | FAME | RCT | CA | 496 | FFR | 509 |  |  |
| Wijns^17^ | 2015 | - | RCT | FFR | 160 | CA | 160 |  |  |
| Wongpraparut^18^ | 2005 | - | prospective | FFR | 57 | CA | 80 |  |  |

**Reference**

1. Andell P, Karlsson S, Mohammad MA, et al. Intravascular Ultrasound Guidance Is Associated With Better Outcome in Patients Undergoing Unprotected Left Main Coronary Artery Stenting Compared With Angiography Guidance Alone. *Circ Cardiovasc Interv* 2017;10(5) doi: 10.1161/CIRCINTERVENTIONS.116.004813

2. Chen SL, Ye F, Zhang JJ, et al. Randomized Comparison of FFR-Guided and Angiography-Guided Provisional Stenting of True Coronary Bifurcation Lesions: The DKCRUSH-VI Trial (Double Kissing Crush Versus Provisional Stenting Technique for Treatment of Coronary Bifurcation Lesions VI). *JACC Cardiovasc Interv* 2015;8(4):536-46. doi: 10.1016/j.jcin.2014.12.221

3. D'Ascenzo F, Iannaccone M, De Filippo O, et al. Optical coherence tomography compared with fractional flow reserve guided approach in acute coronary syndromes: A propensity matched analysis. *Int J Cardiol* 2017;244:54-58. doi: 10.1016/j.ijcard.2017.05.108

4. Davies JE, Sen S, Dehbi HM, et al. Use of the Instantaneous Wave-free Ratio or Fractional Flow Reserve in PCI. *N Engl J Med* 2017;376(19):1824-34. doi: 10.1056/NEJMoa1700445

5. Frohlich GM, Redwood S, Rakhit R, et al. Long-term survival in patients undergoing percutaneous interventions with or without intracoronary pressure wire guidance or intracoronary ultrasonographic imaging: a large cohort study. *JAMA Intern Med* 2014;174(8):1360-6. doi: 10.1001/jamainternmed.2014.1595

6. Gaster AL, Slothuus Skjoldborg U, Larsen J, et al. Continued improvement of clinical outcome and cost effectiveness following intravascular ultrasound guided PCI: insights from a prospective, randomised study. *Heart* 2003;89(9):1043-9.

7. Di Giorgio A, Capodanno D, Ramazzotti V, et al. Optical coherence tomography guided in-stent thrombus removal in patients with acute coronary syndromes. *Int J Cardiovasc Imaging* 2013;29(5):989-96. doi: 10.1007/s10554-013-0191-0

8. Gotberg M, Christiansen EH, Gudmundsdottir IJ, et al. Instantaneous Wave-free Ratio versus Fractional Flow Reserve to Guide PCI. *N Engl J Med* 2017;376(19):1813-23. doi: 10.1056/NEJMoa1616540

9. Huang CL, Jen HL, Huang WP, et al. The Impact of Fractional Flow Reserve-Guided Coronary Revascularization in Patients with Coronary Stenoses of Intermediate Severity. *Acta Cardiol Sin* 2017;33(4):353-61.

10. Jones DA, Rathod KS, Koganti S, et al. Angiography Alone Versus Angiography Plus Optical Coherence Tomography to Guide Percutaneous Coronary Intervention: Outcomes From the Pan-London PCI Cohort. *JACC Cardiovasc Interv* 2018;11(14):1313-21. doi: 10.1016/j.jcin.2018.01.274

11. Mariani J, Jr., Guedes C, Soares P, et al. Intravascular ultrasound guidance to minimize the use of iodine contrast in percutaneous coronary intervention: the MOZART (Minimizing cOntrast utiliZation With IVUS Guidance in coRonary angioplasTy) randomized controlled trial. *JACC Cardiovasc Interv* 2014;7(11):1287-93. doi: 10.1016/j.jcin.2014.05.024

12. Li J, Elrashidi MY, Flammer AJ, et al. Long-term outcomes of fractional flow reserve-guided vs. angiography-guided percutaneous coronary intervention in contemporary practice. *Eur Heart J* 2013;34(18):1375-83. doi: 10.1093/eurheartj/eht005

13. Nam CW, Yoon HJ, Cho YK, et al. Outcomes of percutaneous coronary intervention in intermediate coronary artery disease: fractional flow reserve-guided versus intravascular ultrasound-guided. *JACC Cardiovasc Interv* 2010;3(8):812-7. doi: 10.1016/j.jcin.2010.04.016

14. van Nunen LX, Zimmermann FM, Tonino PA, et al. Fractional flow reserve versus angiography for guidance of PCI in patients with multivessel coronary artery disease (FAME): 5-year follow-up of a randomised controlled trial. *Lancet* 2015;386(10006):1853-60. doi: 10.1016/S0140-6736(15)00057-4

15. Prati F, Di Vito L, Biondi-Zoccai G, et al. Angiography alone versus angiography plus optical coherence tomography to guide decision-making during percutaneous coronary intervention: the Centro per la Lotta contro l'Infarto-Optimisation of Percutaneous Coronary Intervention (CLI-OPCI) study. *EuroIntervention* 2012;8(7):823-9. doi: 10.4244/EIJV8I7A125

16. Tonino PA, De Bruyne B, Pijls NH, et al. Fractional flow reserve versus angiography for guiding percutaneous coronary intervention. *N Engl J Med* 2009;360(3):213-24. doi: 10.1056/NEJMoa0807611

17. Wijns W, Shite J, Jones MR, et al. Optical coherence tomography imaging during percutaneous coronary intervention impacts physician decision-making: ILUMIEN I study. *Eur Heart J* 2015;36(47):3346-55. doi: 10.1093/eurheartj/ehv367

18. Wongpraparut N, Yalamanchili V, Pasnoori V, et al. Thirty-month outcome after fractional flow reserve-guided versus conventional multivessel percutaneous coronary intervention. *Am J Cardiol* 2005;96(7):877-84. doi: 10.1016/j.amjcard.2005.05.040
