# Supplementary material for: How to guide PCI? A network meta-analysis
Source: Medicine (Baltimore). 2020 May 15;99(20):e20168. doi: 10.1097/MD.0000000000020168 (PMC7253719; doi:10.1097/MD.0000000000020168)
Supplement: Supplemental Digital Content [file medi-99-e20168-s019.docx]

**ONLINE SUPPLEMENT**

**How to guide PCI?**

Jun Pang, MD, Liwen Ye, MD, Qingwei Chen, MD, PhD,

**Figures**

Figure 1. Risk of bias assessment.

Figure 2. Flow diagram indicating the disposition of the studies.

Figure 3. Pairwise meta-analysis and heterogeneity comparing different kinds of guidance for PCI on mortality (All). (CA, coronary angiography; FFR, fractional flow reserve; iFR, instantaneous wave-free ratio; IVUS, intravascular ultrasound; OCT, optical coherence tomography)

Figure 4. Pairwise meta-analysis and heterogeneity comparing different kinds of guidance for PCI on mortality (RCT). (CA, coronary angiography; FFR, fractional flow reserve; iFR, instantaneous wave-free ratio; IVUS, intravascular ultrasound; OCT, optical coherence tomography)

Figure 5. Convergence graph of guidance for PCI on mortality (All). (CA, coronary angiography; FFR, fractional flow reserve; iFR, instantaneous wave-free ratio; IVUS, intravascular ultrasound; OCT, optical coherence tomography)

Figure 6. Convergence graph of guidance for PCI on mortality (RCT). (CA, coronary angiography; FFR, fractional flow reserve; iFR, instantaneous wave-free ratio; IVUS, intravascular ultrasound; OCT, optical coherence tomography)

Figure 7. Rank probability plots of the mortality results of the network meta-analysis. As the rank decreases, the bar becomes lighter. (A: All; B: RCT) (CA, coronary angiography; FFR, fractional flow reserve; iFR, instantaneous wave-free ratio; IVUS, intravascular ultrasound; OCT, optical coherence tomography)

Figure 8. Pairwise meta-analysis and heterogeneity comparing different kinds of guidance for PCI on MACEs (All). (CA, coronary angiography; FFR, fractional flow reserve; iFR, instantaneous wave-free ratio; IVUS, intravascular ultrasound; OCT, optical coherence tomography)

Figure 9. Pairwise meta-analysis and heterogeneity comparing different kinds of guidance for PCI on MACEs (RCT). (CA, coronary angiography; FFR, fractional flow reserve; iFR, instantaneous wave-free ratio; IVUS, intravascular ultrasound; OCT, optical coherence tomography)

Figure 10. Convergence graph of guidance for PCI on MACEs (All). (CA, coronary angiography; FFR, fractional flow reserve; iFR, instantaneous wave-free ratio; IVUS, intravascular ultrasound; OCT, optical coherence tomography)

Figure 11. Convergence graph of guidance for PCI on MACEs (RCT). (CA, coronary angiography; FFR, fractional flow reserve; iFR, instantaneous wave-free ratio; IVUS, intravascular ultrasound; OCT, optical coherence tomography)

Figure 12. Rank probability plots of the MACEs results of the network meta-analysis. As the rank decreases, the bar becomes lighter. (A: All; B: RCT) (CA, coronary angiography; FFR, fractional flow reserve; iFR, instantaneous wave-free ratio; IVUS, intravascular ultrasound; OCT, optical coherence tomography)

Figure 13. Pairwise meta-analysis and heterogeneity comparing different kinds of guidance for PCI on MI (All). (CA, coronary angiography; FFR, fractional flow reserve; iFR, instantaneous wave-free ratio; IVUS, intravascular ultrasound; OCT, optical coherence tomography)

Figure 14. Pairwise meta-analysis and heterogeneity comparing different kinds of guidance for PCI on MI (RCT). (CA, coronary angiography; FFR, fractional flow reserve; iFR, instantaneous wave-free ratio; IVUS, intravascular ultrasound; OCT, optical coherence tomography)

Figure 15. Convergence graph of guidance for PCI on MI (All). (CA, coronary angiography; FFR, fractional flow reserve; iFR, instantaneous wave-free ratio; IVUS, intravascular ultrasound; OCT, optical coherence tomography)

Figure 16. Convergence graph of guidance for PCI on MI (RCT). (CA, coronary angiography; FFR, fractional flow reserve; iFR, instantaneous wave-free ratio; IVUS, intravascular ultrasound; OCT, optical coherence tomography)

Figure 17. Rank probability plots of the MI results of the network meta-analysis. As the rank decreases, the bar becomes lighter. (A: All; B: RCT) (CA, coronary angiography; FFR, fractional flow reserve; iFR, instantaneous wave-free ratio; IVUS, intravascular ultrasound; OCT, optical coherence tomography)

**Tables**

Table 1. Baseline characteristics of the trials that were included in the network meta-analysis. (CA, coronary angiography; FFR, fractional flow reserve; iFR, instantaneous wave-free ratio; IVUS, intravascular ultrasound; OCT, optical coherence tomography)

**Figures**


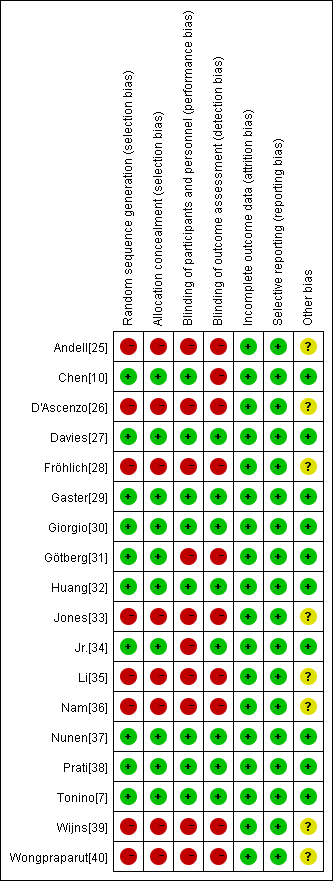
**Figure 1-A.** Risk of bias summary:

judgements about each bias item for

each study.

**Figure 1-B.** Risk of bias graph: review authors' judgements (Low, Unclear and High) about each risk of bias item presented as percentages across all included studies.


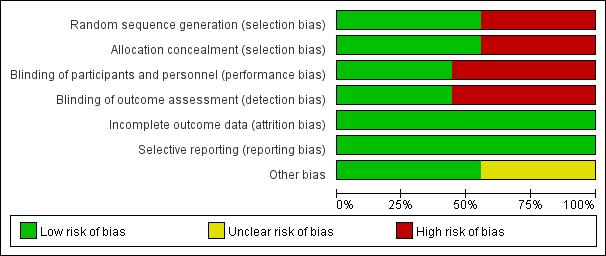


Figure 2. Flow diagram indicating the disposition of the studies.


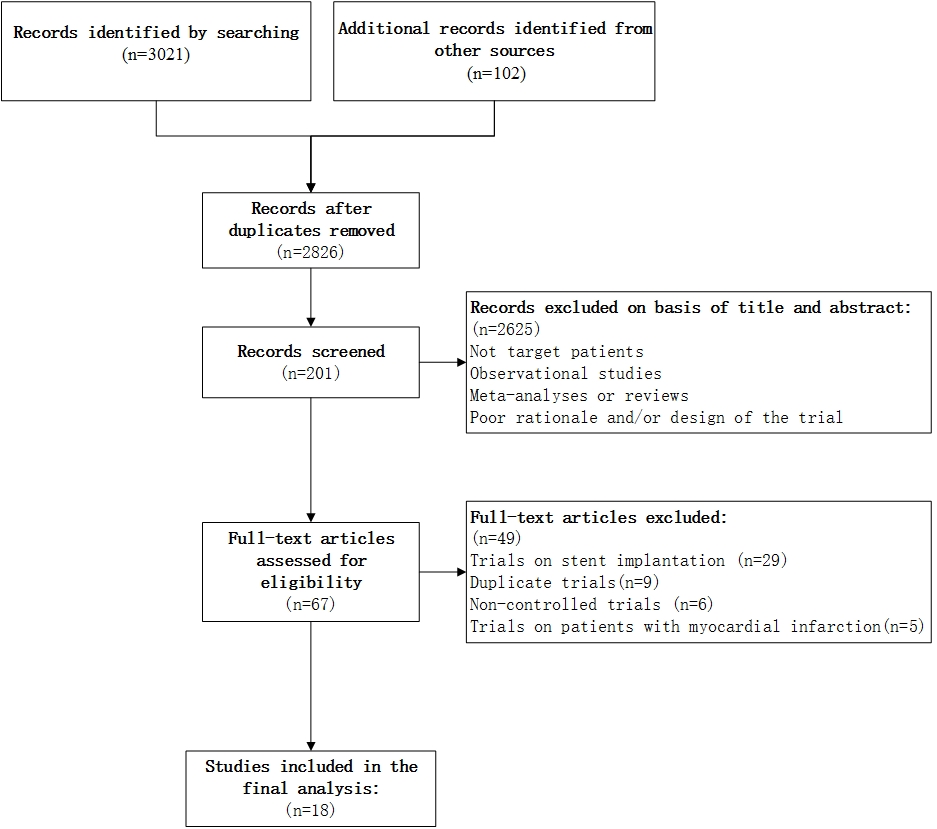


Figure 3. Pairwise meta-analysis and heterogeneity comparing different kinds of guidance for PCI on mortality (All). (CA, coronary angiography; FFR, fractional flow reserve; iFR, instantaneous wave-free ratio; IVUS, intravascular ultrasound; OCT, optical coherence tomography)


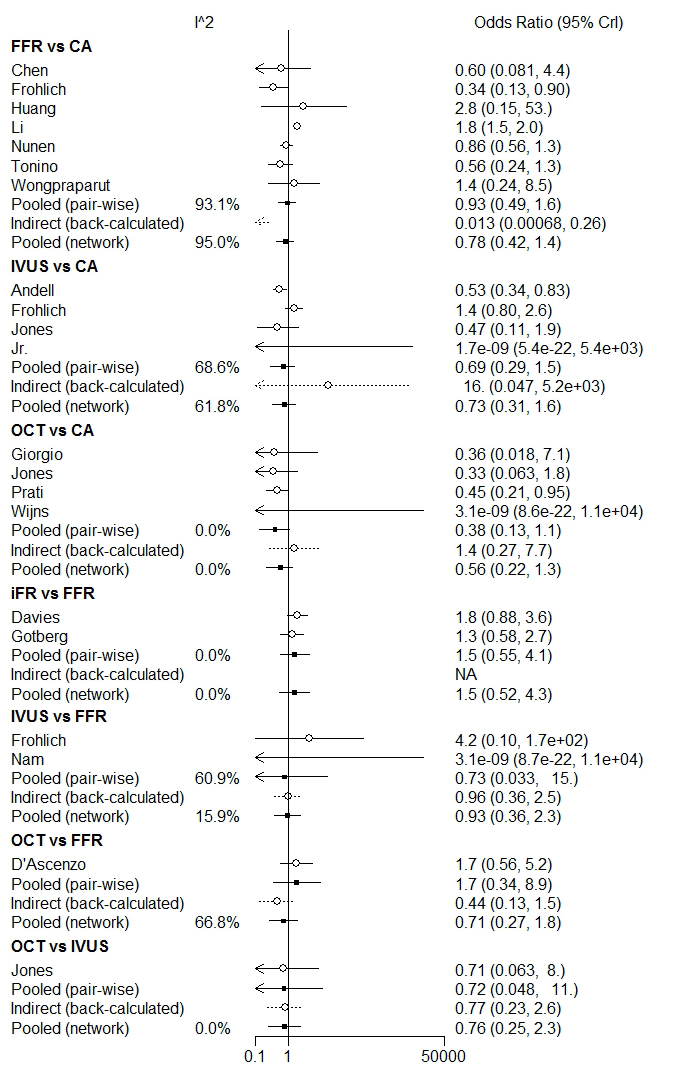


Figure 4. Pairwise meta-analysis and heterogeneity comparing different kinds of guidance for PCI on mortality (RCT). (CA, coronary angiography; FFR, fractional flow reserve; iFR, instantaneous wave-free ratio; IVUS, intravascular ultrasound; OCT, optical coherence tomography)


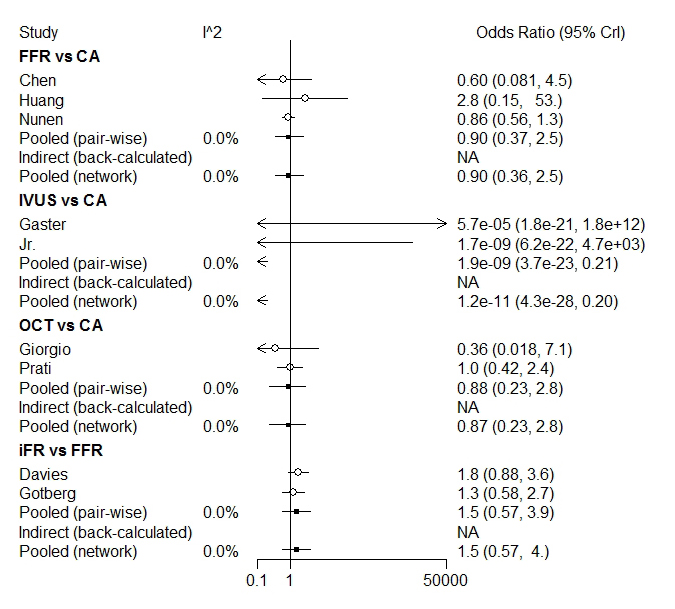


Figure 5. Convergence graph of guidance for PCI on mortality (All). (CA, coronary angiography; FFR, fractional flow reserve; iFR, instantaneous wave-free ratio; IVUS, intravascular ultrasound; OCT, optical coherence tomography)


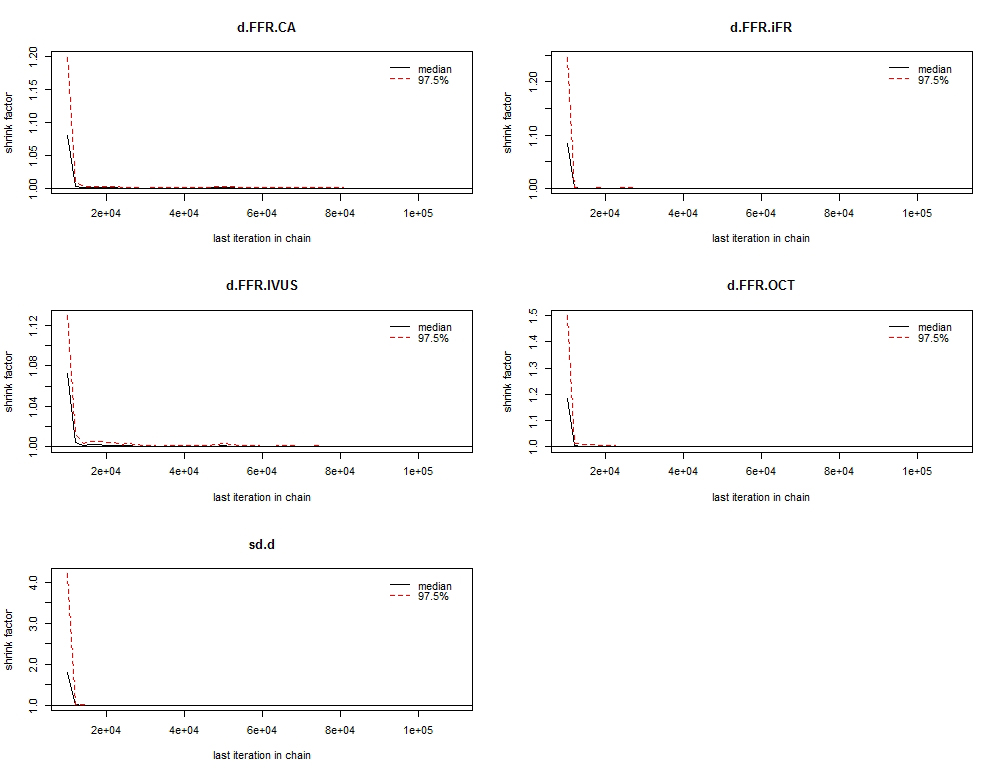


Figure 6. Convergence graph of guidance for PCI on mortality (RCT). (CA, coronary angiography; FFR, fractional flow reserve; iFR, instantaneous wave-free ratio; IVUS, intravascular ultrasound; OCT, optical coherence tomography)


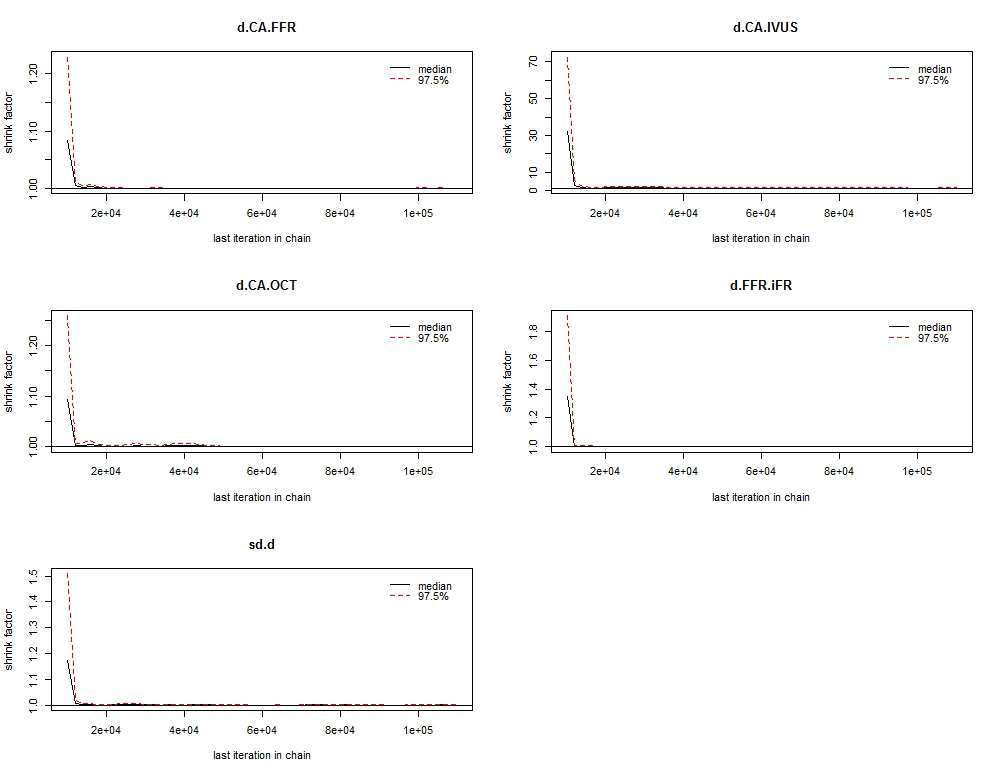


Figure 7. Rank probability plots of the mortality results of the network meta-analysis. As the rank decreases, the bar becomes lighter. (A: All; B: RCT) (CA, coronary angiography; FFR, fractional flow reserve; iFR, instantaneous wave-free ratio; IVUS, intravascular ultrasound; OCT, optical coherence tomography)


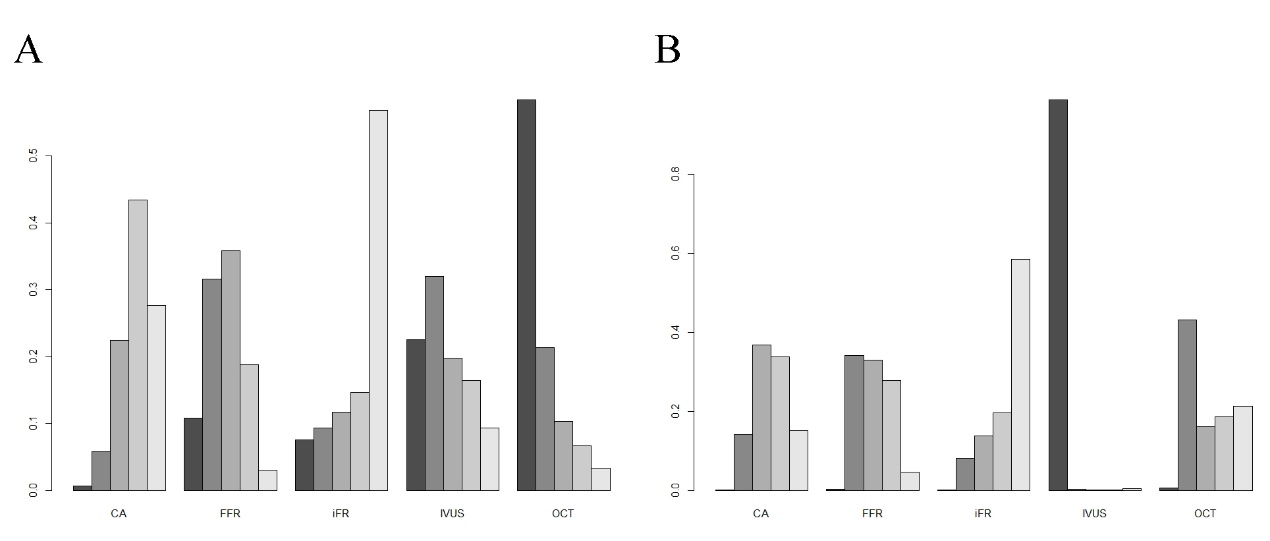


Figure 8. Pairwise meta-analysis and heterogeneity comparing different kinds of guidance for PCI on MACEs (All). (CA, coronary angiography; FFR, fractional flow reserve; iFR, instantaneous wave-free ratio; IVUS, intravascular ultrasound; OCT, optical coherence tomography)


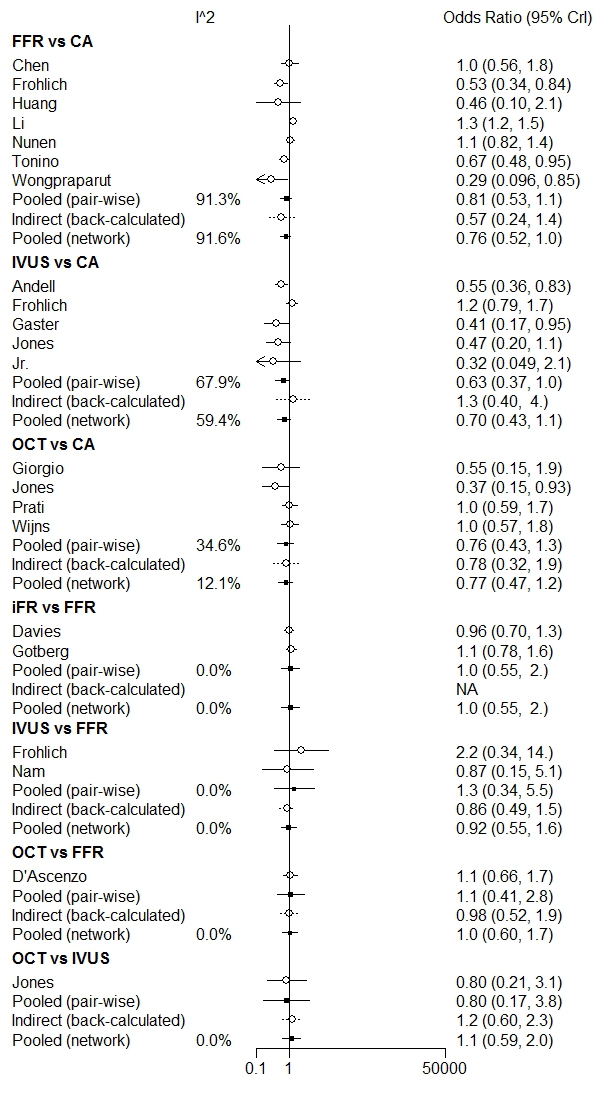


Figure 9. Pairwise meta-analysis and heterogeneity comparing different kinds of guidance for PCI on MACEs (RCT). (CA, coronary angiography; FFR, fractional flow reserve; iFR, instantaneous wave-free ratio; IVUS, intravascular ultrasound; OCT, optical coherence tomography)


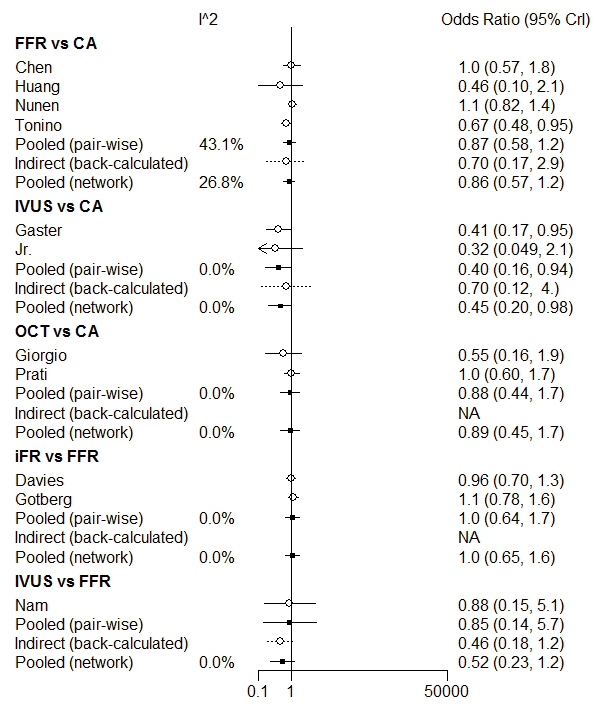


Figure 10. Convergence graph of guidance for PCI on MACEs (All). (CA, coronary angiography; FFR, fractional flow reserve; iFR, instantaneous wave-free ratio; IVUS, intravascular ultrasound; OCT, optical coherence tomography)


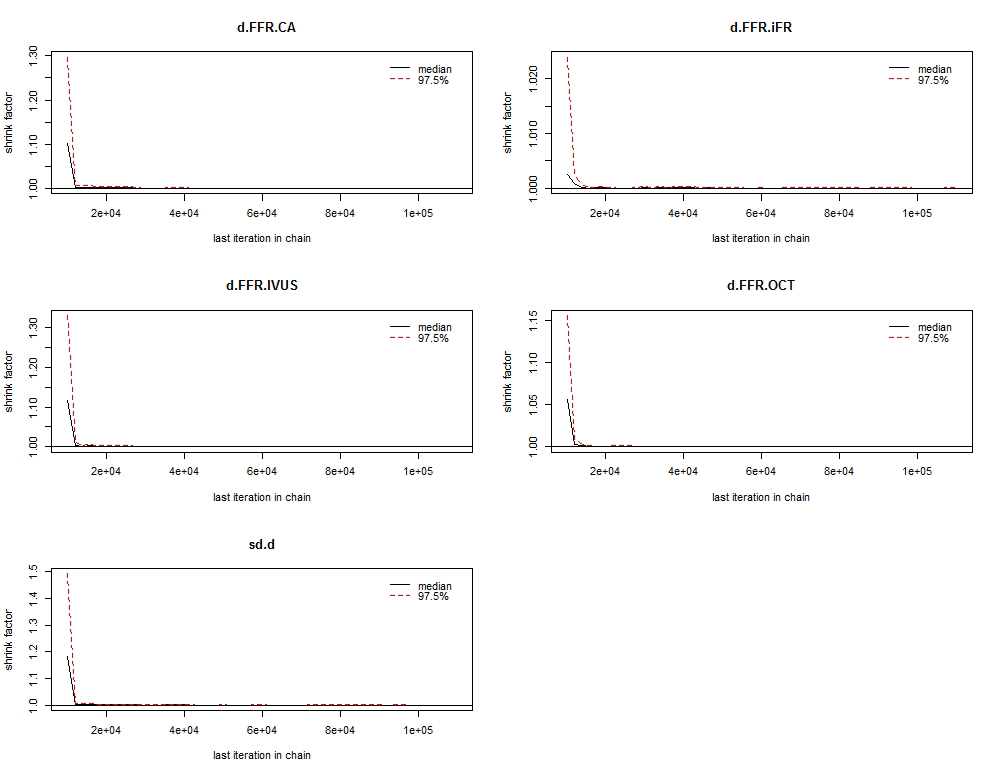


Figure 11. Convergence graph of guidance for PCI on MACEs (RCT). (CA, coronary angiography; FFR, fractional flow reserve; iFR, instantaneous wave-free ratio; IVUS, intravascular ultrasound; OCT, optical coherence tomography)


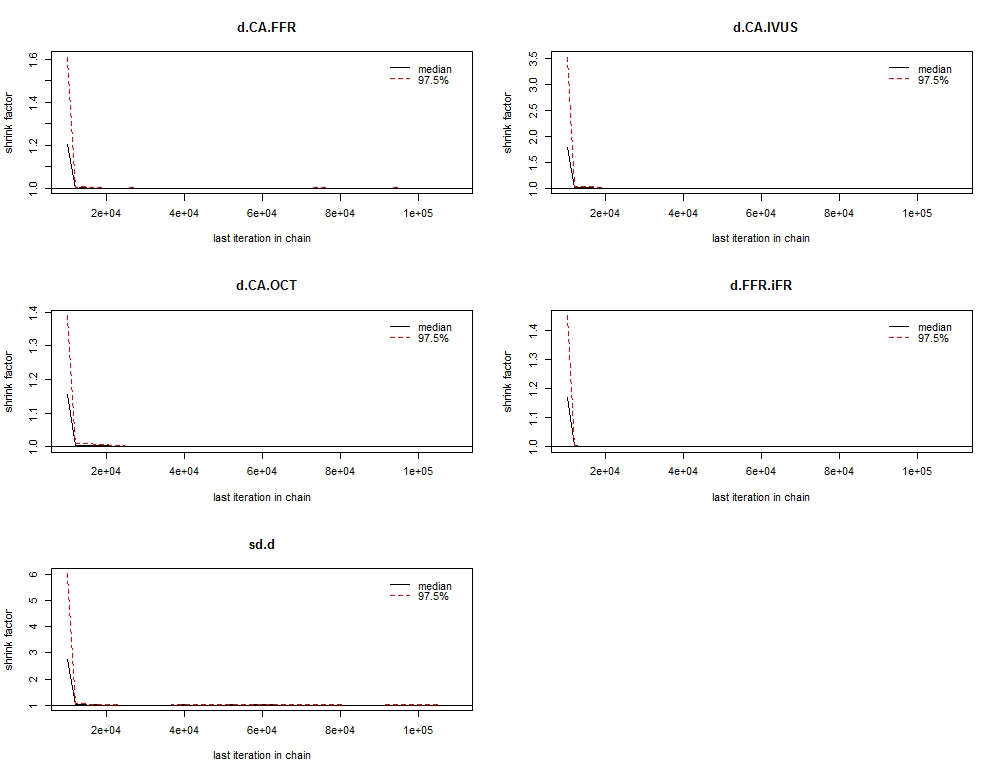


Figure 12. Rank probability plots of the MACEs results of the network meta-analysis. As the rank decreases, the bar becomes lighter. (A: All; B: RCT) (CA, coronary angiography; FFR, fractional flow reserve; iFR, instantaneous wave-free ratio; IVUS, intravascular ultrasound; OCT, optical coherence tomography)


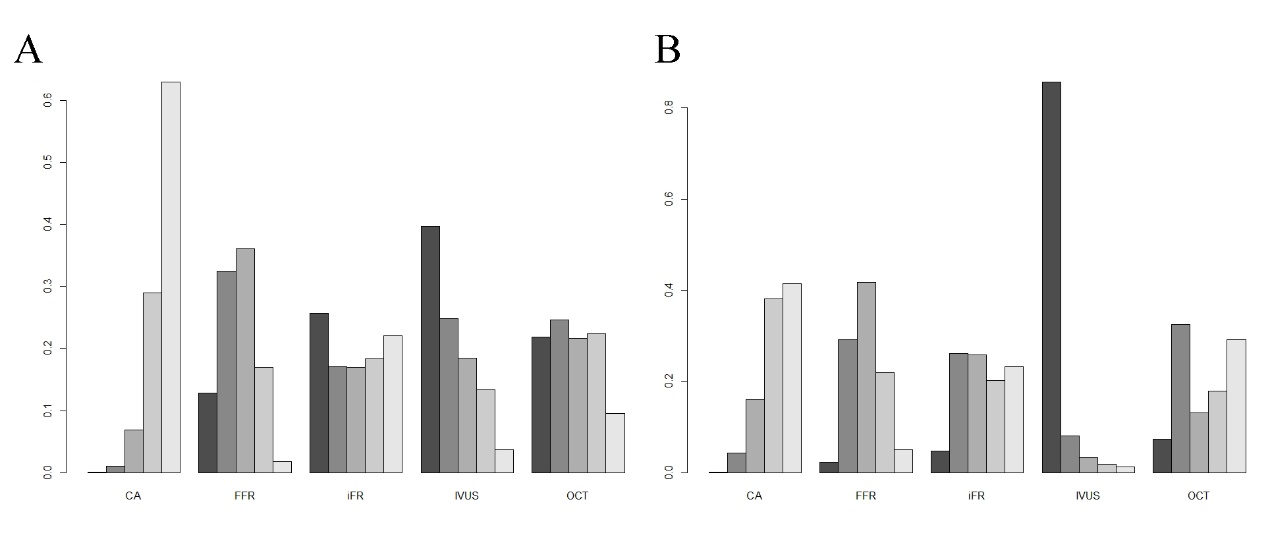


Figure 13. Pairwise meta-analysis and heterogeneity comparing different kinds of guidance for PCI on MI (All). (CA, coronary angiography; FFR, fractional flow reserve; iFR, instantaneous wave-free ratio; IVUS, intravascular ultrasound; OCT, optical coherence tomography)


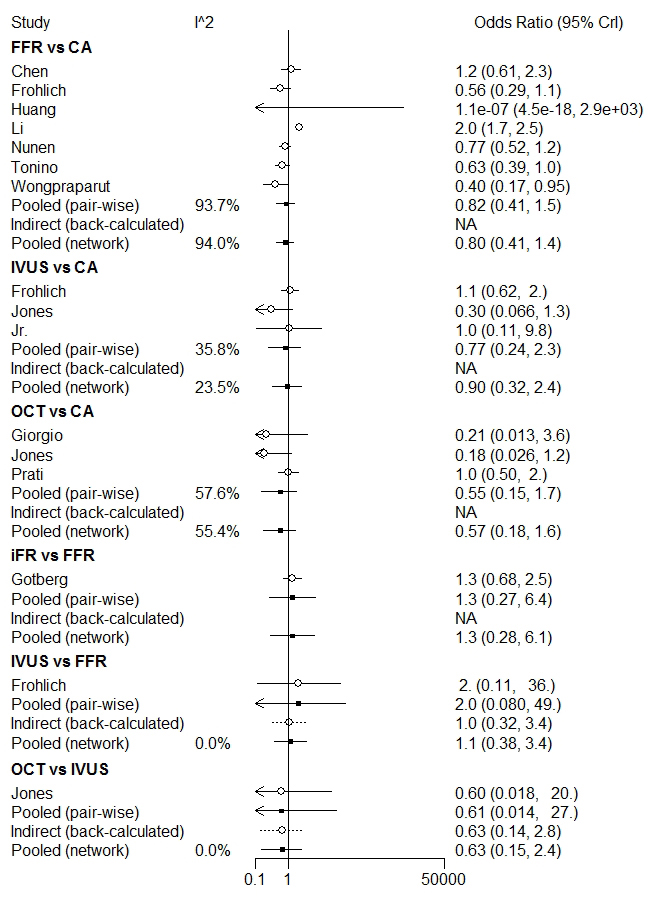


Figure 14. Pairwise meta-analysis and heterogeneity comparing different kinds of guidance for PCI on MI (RCT). (CA, coronary angiography; FFR, fractional flow reserve; iFR, instantaneous wave-free ratio; IVUS, intravascular ultrasound; OCT, optical coherence tomography)


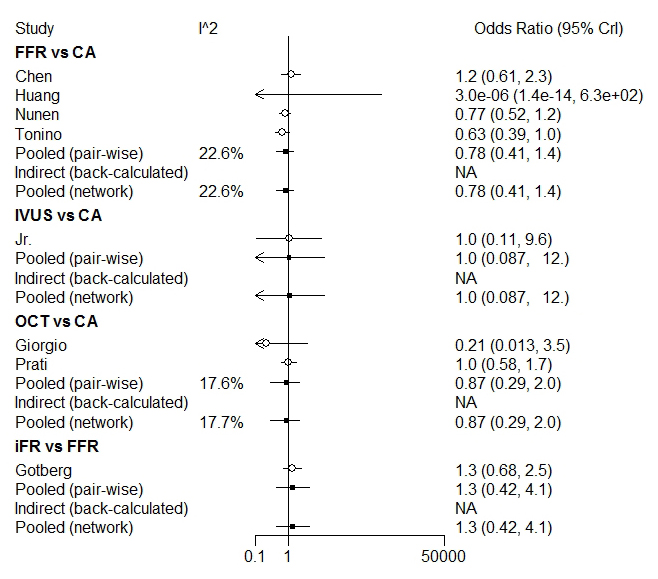


Figure 15. Convergence graph of guidance for PCI on MI (All). (CA, coronary angiography; FFR, fractional flow reserve; iFR, instantaneous wave-free ratio; IVUS, intravascular ultrasound; OCT, optical coherence tomography)


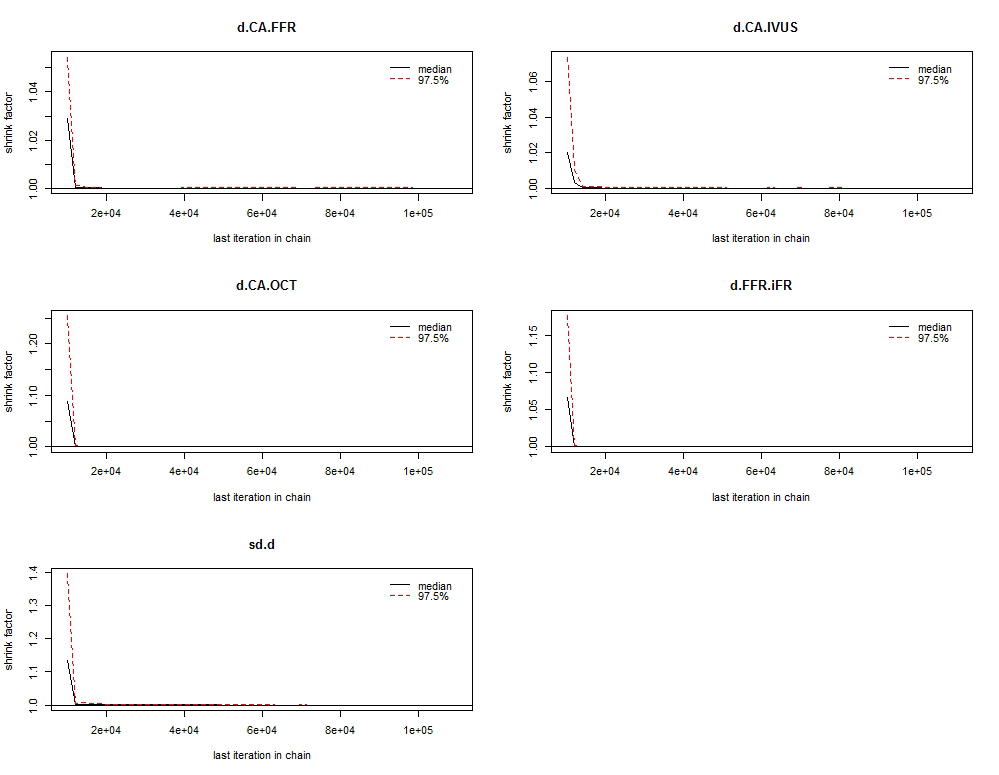


Figure 16. Convergence graph of guidance for PCI on MI (RCT). (CA, coronary angiography; FFR, fractional flow reserve; iFR, instantaneous wave-free ratio; IVUS, intravascular ultrasound; OCT, optical coherence tomography)


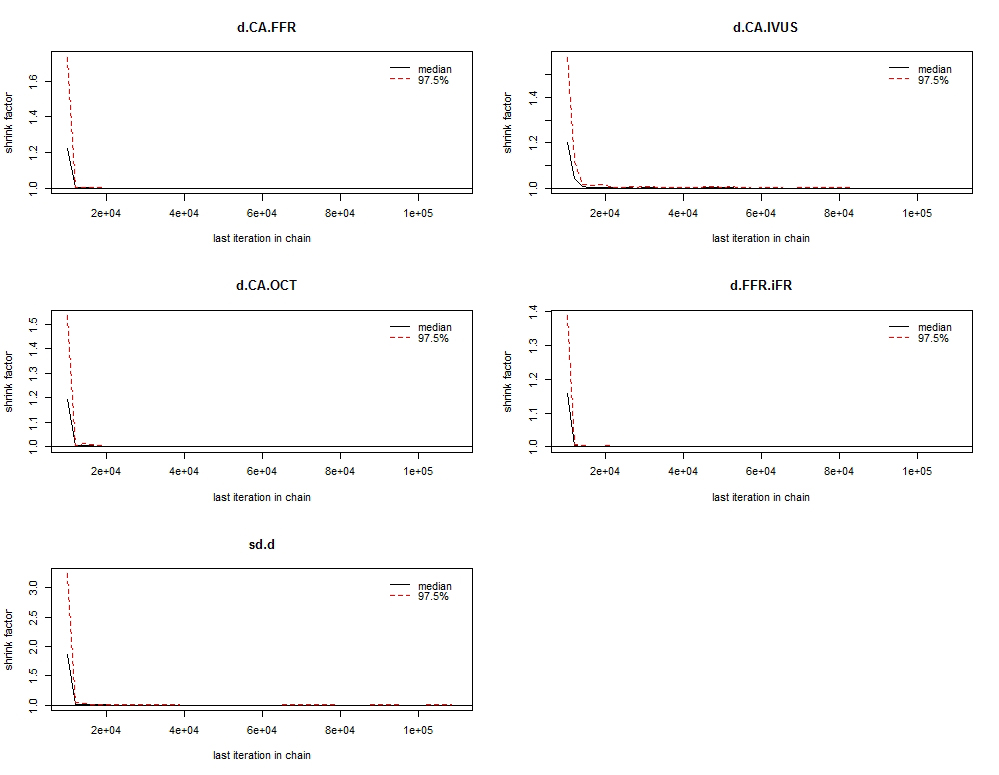


Figure 17. Rank probability plots of the MI results of the network meta-analysis. As the rank decreases, the bar becomes lighter. (A: All; B: RCT) (CA, coronary angiography; FFR, fractional flow reserve; iFR, instantaneous wave-free ratio; IVUS, intravascular ultrasound; OCT, optical coherence tomography)


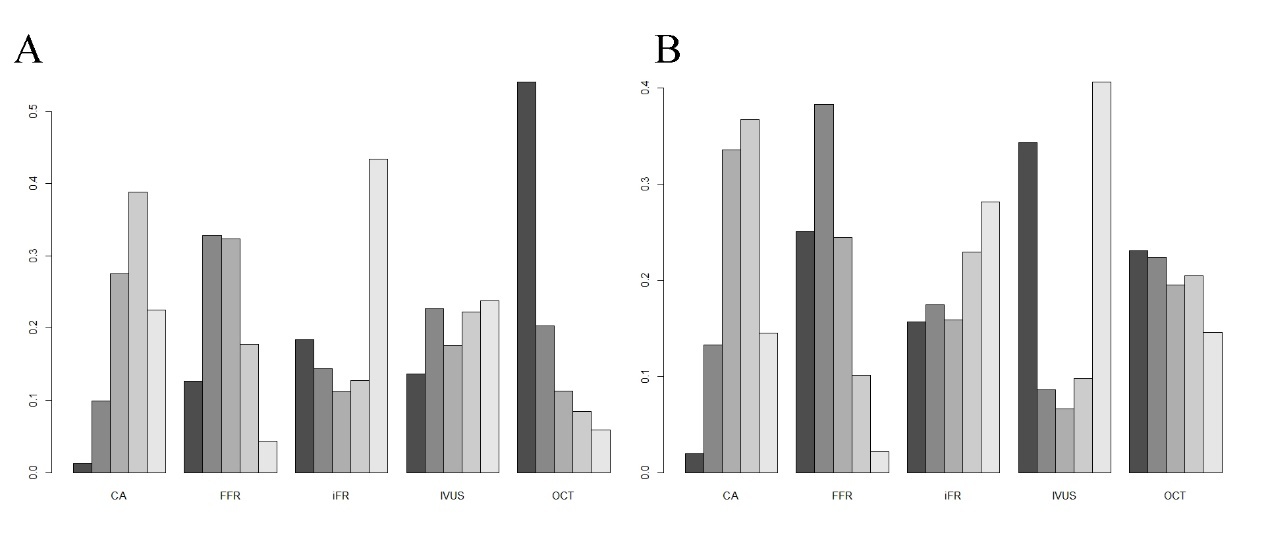


Table 1. Baseline characteristics of the trials that were included in the network meta-analysis. (CA, coronary angiography; FFR, fractional flow reserve; iFR, instantaneous wave-free ratio; IVUS, intravascular ultrasound; OCT, optical coherence tomography)

| Author | Year | Study | RCT | Method1 | Size1 | Method2 | Size2 | Mehod3 | Size3 |
| --- | --- | --- | --- | --- | --- | --- | --- | --- | --- |
| Andell^1^ | 2017 | - | prospective | CA | 340 | IVUS | 340 |  |  |
| Chen^2^ | 2015 | ILUMIEN I | prospective | CA | 137 | OCT | 165 |  |  |
| D'Ascenzo^3^ | 2017 | - | prospective | OCT | 285 | FFR | 355 |  |  |
| Davies^4^ | 2017 | DEFINE-FLAIR | RCT | iFR | 1147 | FFR | 1179 |  |  |
| Fröhlich^5^ | 2014 | - | retrospective | CA | 37090 | FFR | 2767 | IVUS | 1831 |
| Gaster^6^ | 2003 | MUSIC | RCT | IVUS | 54 | CA | 54 |  |  |
| Giorgio^7^ | 2013 | - | RCT | OCT | 40 | CA | 40 |  |  |
| Götberg^8^ | 2017 | iFR-SWEDEHEART | RCT | iFR | 1012 | FFR | 1007 |  |  |
| Huang^9^ | 2017 | - | RCT | FFR | 101 | CA | 105 |  |  |
| Jones^10^ | 2018 | Pan-London | retrospective | CA | 1134 | OCT | 1134 | IVUS | 1125 |
| Jr.^11^ | 2015 | MOZART | RCT | IVUS | 41 | CA | 42 |  |  |
| Li^12^ | 2012 | - | retrospective | CA | 6268 | FFR | 1090 |  |  |
| Nam^13^ | 2010 | - | retrospective | FFR | 83 | IVUS | 94 |  |  |
| Nunen^14^ | 2015 | FAME | RCT | CA | 496 | FFR | 509 |  |  |
| Prati^15^ | 2012 | CLI-OPCI | RCT | OCT | 335 | CA | 335 |  |  |
| Tonino^16^ | 2009 | FAME | RCT | CA | 496 | FFR | 509 |  |  |
| Wijns^17^ | 2015 | - | RCT | FFR | 160 | CA | 160 |  |  |
| Wongpraparut^18^ | 2005 | - | prospective | FFR | 57 | CA | 80 |  |  |

**Reference**

1. Andell P, Karlsson S, Mohammad MA, et al. Intravascular Ultrasound Guidance Is Associated With Better Outcome in Patients Undergoing Unprotected Left Main Coronary Artery Stenting Compared With Angiography Guidance Alone. *Circ Cardiovasc Interv* 2017;10(5) doi: 10.1161/CIRCINTERVENTIONS.116.004813

2. Chen SL, Ye F, Zhang JJ, et al. Randomized Comparison of FFR-Guided and Angiography-Guided Provisional Stenting of True Coronary Bifurcation Lesions: The DKCRUSH-VI Trial (Double Kissing Crush Versus Provisional Stenting Technique for Treatment of Coronary Bifurcation Lesions VI). *JACC Cardiovasc Interv* 2015;8(4):536-46. doi: 10.1016/j.jcin.2014.12.221

3. D'Ascenzo F, Iannaccone M, De Filippo O, et al. Optical coherence tomography compared with fractional flow reserve guided approach in acute coronary syndromes: A propensity matched analysis. *Int J Cardiol* 2017;244:54-58. doi: 10.1016/j.ijcard.2017.05.108

4. Davies JE, Sen S, Dehbi HM, et al. Use of the Instantaneous Wave-free Ratio or Fractional Flow Reserve in PCI. *N Engl J Med* 2017;376(19):1824-34. doi: 10.1056/NEJMoa1700445

5. Frohlich GM, Redwood S, Rakhit R, et al. Long-term survival in patients undergoing percutaneous interventions with or without intracoronary pressure wire guidance or intracoronary ultrasonographic imaging: a large cohort study. *JAMA Intern Med* 2014;174(8):1360-6. doi: 10.1001/jamainternmed.2014.1595

6. Gaster AL, Slothuus Skjoldborg U, Larsen J, et al. Continued improvement of clinical outcome and cost effectiveness following intravascular ultrasound guided PCI: insights from a prospective, randomised study. *Heart* 2003;89(9):1043-9.

7. Di Giorgio A, Capodanno D, Ramazzotti V, et al. Optical coherence tomography guided in-stent thrombus removal in patients with acute coronary syndromes. *Int J Cardiovasc Imaging* 2013;29(5):989-96. doi: 10.1007/s10554-013-0191-0

8. Gotberg M, Christiansen EH, Gudmundsdottir IJ, et al. Instantaneous Wave-free Ratio versus Fractional Flow Reserve to Guide PCI. *N Engl J Med* 2017;376(19):1813-23. doi: 10.1056/NEJMoa1616540

9. Huang CL, Jen HL, Huang WP, et al. The Impact of Fractional Flow Reserve-Guided Coronary Revascularization in Patients with Coronary Stenoses of Intermediate Severity. *Acta Cardiol Sin* 2017;33(4):353-61.

10. Jones DA, Rathod KS, Koganti S, et al. Angiography Alone Versus Angiography Plus Optical Coherence Tomography to Guide Percutaneous Coronary Intervention: Outcomes From the Pan-London PCI Cohort. *JACC Cardiovasc Interv* 2018;11(14):1313-21. doi: 10.1016/j.jcin.2018.01.274

11. Mariani J, Jr., Guedes C, Soares P, et al. Intravascular ultrasound guidance to minimize the use of iodine contrast in percutaneous coronary intervention: the MOZART (Minimizing cOntrast utiliZation With IVUS Guidance in coRonary angioplasTy) randomized controlled trial. *JACC Cardiovasc Interv* 2014;7(11):1287-93. doi: 10.1016/j.jcin.2014.05.024

12. Li J, Elrashidi MY, Flammer AJ, et al. Long-term outcomes of fractional flow reserve-guided vs. angiography-guided percutaneous coronary intervention in contemporary practice. *Eur Heart J* 2013;34(18):1375-83. doi: 10.1093/eurheartj/eht005

13. Nam CW, Yoon HJ, Cho YK, et al. Outcomes of percutaneous coronary intervention in intermediate coronary artery disease: fractional flow reserve-guided versus intravascular ultrasound-guided. *JACC Cardiovasc Interv* 2010;3(8):812-7. doi: 10.1016/j.jcin.2010.04.016

14. van Nunen LX, Zimmermann FM, Tonino PA, et al. Fractional flow reserve versus angiography for guidance of PCI in patients with multivessel coronary artery disease (FAME): 5-year follow-up of a randomised controlled trial. *Lancet* 2015;386(10006):1853-60. doi: 10.1016/S0140-6736(15)00057-4

15. Prati F, Di Vito L, Biondi-Zoccai G, et al. Angiography alone versus angiography plus optical coherence tomography to guide decision-making during percutaneous coronary intervention: the Centro per la Lotta contro l'Infarto-Optimisation of Percutaneous Coronary Intervention (CLI-OPCI) study. *EuroIntervention* 2012;8(7):823-9. doi: 10.4244/EIJV8I7A125

16. Tonino PA, De Bruyne B, Pijls NH, et al. Fractional flow reserve versus angiography for guiding percutaneous coronary intervention. *N Engl J Med* 2009;360(3):213-24. doi: 10.1056/NEJMoa0807611

17. Wijns W, Shite J, Jones MR, et al. Optical coherence tomography imaging during percutaneous coronary intervention impacts physician decision-making: ILUMIEN I study. *Eur Heart J* 2015;36(47):3346-55. doi: 10.1093/eurheartj/ehv367

18. Wongpraparut N, Yalamanchili V, Pasnoori V, et al. Thirty-month outcome after fractional flow reserve-guided versus conventional multivessel percutaneous coronary intervention. *Am J Cardiol* 2005;96(7):877-84. doi: 10.1016/j.amjcard.2005.05.040
